# Multi-stage optimization strategy based on contextual analysis to create M-health components for case management model in breast cancer transitional care: the CMBM study as an example

**DOI:** 10.1186/s12912-024-02049-x

**Published:** 2024-06-06

**Authors:** Hong Chengang, Wang Liping, Wang Shujin, Chen Chen, Yang Jiayue, Lu Jingjing, Hua Shujie, Wu Jieming, Yao Liyan, Zeng Ni, Chu Jinhui, Sun Jiaqi

**Affiliations:** School of Nursing, Hangzhou Normal University, Hangzhou City, Zhejiang Province 311100 China

**Keywords:** Multi-stage optimization strategy, M-health, User-centered design, Contextual analysis, Breast cancer patients, Case management, Transitional care

## Abstract

**Background:**

None of the early M-Health applications are designed for case management care services. This study aims to describe the process of developing a M-health component for the case management model in breast cancer transitional care and to highlight methods for solving the common obstacles faced during the application of M-health nursing service.

**Methods:**

We followed a four-step process: (a) Forming a cross-functional interdisciplinary development team containing two sub-teams, one for content development and the other for software development. (b) Applying self-management theory as the theoretical framework to develop the M-health application, using contextual analysis to gain a comprehensive understanding of the case management needs of oncology nursing specialists and the supportive care needs of out-of-hospital breast cancer patients. We validated the preliminary concepts of the framework and functionality of the M-health application through multiple interdisciplinary team discussions. (c) Adopting a multi-stage optimization strategy consisting of three progressive stages: screening, refining, and confirmation to develop and continually improve the WeChat mini-programs. (d) Following the user-centered principle throughout the development process and involving oncology nursing specialists and breast cancer patients at every stage.

**Results:**

Through a continuous, iterative development process and rigorous testing, we have developed patient-end and nurse-end program for breast cancer case management. The patient-end program contains four functional modules: “Information”, “Interaction”, “Management”, and “My”, while the nurse-end program includes three functional modules: “Consultation”, “Management”, and “My”. The patient-end program scored 78.75 on the System Usability Scale and showed a 100% task passing rate, indicating that the programs were easy to use.

**Conclusions:**

Based on the contextual analysis, multi-stage optimization strategy, and interdisciplinary team work, a WeChat mini-program has been developed tailored to the requirements of the nurses and patients. This approach leverages the expertise of professionals from multiple disciplines to create effective and evidence-based solutions that can improve patient outcomes and quality of care.

**Supplementary Information:**

The online version contains supplementary material available at 10.1186/s12912-024-02049-x.

## Background

Female breast cancer is the second leading cause of global cancer incidence in 2022, with an estimated 2.3 million new cases, representing 11.6% of all cancer cases [1]. Due to surgical trauma, side effects of drugs, fear of the recurrence or metastasis of breast cancer, changes in female characteristics, and lack of knowledge, patients with breast cancer frequently experience a series of physical and psychological health problems [2–6]. These health problems seriously affected patients’ life and work [7, 8]. At present, community nursing in China is still in the developing stage, and the oncology specialty nursing service capacity of community nurses is not enough to deal with the health problems of breast cancer patients. It made continuous care for out-of-hospital breast cancer patients a weak link in the Chinese oncology nursing service system.

Nowadays, case management is employed to manage health problems for out-of-hospital breast cancer patients worldwide [9–15]. Case management involves regular telephone follow-ups and home visits by case management nurses to provide educational support to patients, thereby ensuring uninterrupted continuity of care [16, 17]. The home visits and organization of patient information required for case management tasks consume a significant amount of time, manpower, and material resources [17]. In China, case management services are primarily undertaken by oncology nursing specialists from tertiary hospitals in their spare time [18]. However, the shortage of nurses has consistently been one of the major challenges facing the nursing industry in China, especially in tertiary hospitals [19]. Consequently, the implementation and promotion of case management in China also face great difficulties in reality [20].

The Global Observatory for eHealth (GOe) of the World Health Organization (WHO) defines mobile health (M-Health) as “medical and public health practice supported by mobile devices, such as mobile phones, patient monitoring devices, personal digital assistants (PDAs), and other wireless devices” [21, 22]. With the development of digital technology and the COVID-19 pandemic in 2019, M-Health applications were further integrated into healthcare services, which increased the demand for M-Health applications in turn [23, 24]. Compared with the traditional health service model, M-Health service model has the advantages of high-level informatization, fast response speed, freedom from time and location constraints, and resource-saving, etc. In the context of limited nursing human resources, M-Health service provides a new solution for the case management of out-of-hospital breast cancer patients [23, 25, 26].

Researchers have developed a range of M-Health applications targeting breast cancer patients. To our knowledge, none of these developed M-Health applications are designed for case management nursing services.

Early M-Health applications were mostly designed for single interventional goals, such as health education, medication compliance, self-monitoring, etc. Larsen et al. applied a M-Health application to monitor and adjust the dosage of oral chemotherapy drugs in breast cancer patients, and the results suggested that the treatment adherence was effectively improved [27]. Heo and his team successfully promoted self-breast-examination behavior in women under 30 years old using a M-Health application [28]. Mccarrol carried out a M-Health diet and exercise intervention in overweight breast cancer patients and found that the weight, BMI, and waist circumference of the intervention group decreased after one month [29]. Smith’s team found that their application promoted the adoption of healthy diet and exercise behaviors among breast cancer patients [30]. The application designed by Eden et al. enhanced the ability of breast cancer patients receiving chemotherapy to recognize adverse drug reactions [31]. Keohane and colleagues designed a health educational application based on the best practices and it proved effective in improving breast cancer-related knowledge [32]. The guideline-based M-Health application developed by Eden et al. optimized breast cancer patients’ individualized health decision-making regarding mammography [33].

With the progress of computer technology and the emphasis on physical and mental rehabilitation of breast cancer patients, some universities [34, 35] in China have separately developed M-Health applications for comprehensive health management, which provide access to online communication, health education, and expert consultation.

Analyzing these developed applications deeply, three factors could be found that hindered the promotion of applications in real life. Firstly, the developing procedure usually lacks contextual analysis based on the actual usage context during the design phase. Secondly, there is a lack of consistent and long-term monitoring and operation staff in the subsequent program implementation. These factors may be the main reasons why many M-Health applications face difficulties in promotion and continuous operation after the research phase. Furthermore, as applications need to be installed on patients’ smartphones, certain hardware requirements, such as memory, may also pose restrict the adoption of M-Health applications to some extent.

In order to meet the needs of supportive care for out-of-hospital breast cancer patients and the needs of case management for oncology nurse specialists, we formed a multidisciplinary research team and collaboratively developed a WeChat mini-program for breast cancer case management in the CMBM ***(M-health for case management model in breast cancer transitional care)*** project. WeChat is chosen as the program development platform based on the following considerations. Firstly, WeChat is the most popular and widely used social software in China. As of December 31, 2020, the monthly active users of WeChat have exceeded 1.2 billion, and the daily active users of WeChat mini-programs exceeded 450 million [36]. Secondly, users can access and use the services of the mini-program directly within the WeChat platform, without the need to download or install additional mobile applications. This reduces the hardware requirements for software applications. The above two factors allow for a positive user experience and a realistic foundation for software promotion.

The purpose of this study is to describe the process of developing a tailored M-health component for the case management model in breast cancer transitional care and to highlight methods for solving the common obstacles faced during the application of M-health nursing service.

## Methods and results

The development process was conducted in four steps: (a) An interdisciplinary development team was formed, consisting of two sub-teams dedicated to content and software development. (b) Using the self-management theory as the theoretical framework, contextual analysis was used to understand the case management needs of oncology nursing specialists and the supportive care needs of out-of-hospital breast cancer patients. Through iterative discussion within the interdisciplinary team, the preliminary conception of the application framework and function was formed. (c) A multi-stage optimization strategy was adopted to develop and regularly update the WeChat mini-programs, including three stages (screening, refining, and confirming). (d) During the entire development process, a user-centered principle was followed with the involvement of oncology nursing specialists and breast cancer patients, including development, testing, and iterative development phases.

## The interdisciplinary team

An important prerequisite for developing M-health applications is the formation of an interdisciplinary development team. We built a multidisciplinary team consisting of researchers, oncology nursing specialists, and software developers. Each team member brought their expertise from their respective fields, and all individuals were considered members of the same team rather than separate participants with a common goal.

Two sub-teams were established, one responsible for content development, and the other for software development. The content development team consisted of researchers and six senior breast oncology nursing specialists with bachelor’s degrees and over 10 years of clinical experience. Their work included contextual analysis, functional framework design, and content review of the “Information” module. The software development team included researchers and experienced software developers. Their tasks involved developing the mini-program based on the functional framework and requirements designed by the content development team.

The development team used contextual analysis to identify the actual usage needs of two target groups for the mini-program: oncologist nurse specialists and out-of-hospital breast cancer patients.

### Involvement of oncology nursing specialists and breast cancer patients following user-centered design principle

Since the oncology nursing specialists and breast cancer patients are targeted users of the mini-program, the two groups fully participated in the development according to the user-centered principle. Nursing specialists who in charge of case management were interviewed about the preliminary functional framework of the mini-program. The interview results are presented in the section “Driving the Development Process via the Contextual Analysis Findings.” Semi-structured in-depth interviews were conducted in the testing and iteration stage to gain user feedback from nursing specialists to improve the applicability and usability of the mini-program. The interview guide can be found in the supplementary material.

Breast cancer patients fully engaged in the three developing phases (Screening, Refining, and Confirming). In the Screening Phase, since the self-management theory was selected as the theoretical framework, the supportive care needs of out-of-hospital breast cancer patients were explored, and the functional framework of the mini-program was constructed accordingly. In the Refining Phase, patients were invited to evaluate the usability and practicality of the mini-program through system tests and semi-structured in-depth interviews. The results of the system test are presented in the Results of System Test section. The feedback from interviews and corresponding iterative updates are listed in Table 1. In the Confirming Phase, our research team is conducting clinical trials in out-of-hospital breast cancer patients to find out the actual effect of the mini-program on recovery.

**Table 1:** The feedback and iterative updates

Shortcomings of version 1.0 mini-program	Iterative updates
“Information”: (1) The order of symptom labels in the “Physiological field” of “Breast Cancer Rehabilitation” is not appropriate. (2) The search box pattern is too small to use.	(1) Labels such as “Surgical incision” and “Arm function exercise” have been moved to the front. (2) Resolved.
“Interaction”: When the nurse and patient receive messages, no message reminder pops up on the WeChat interface timely.	Resolved.
“Management”: No clock-in reminder function.	Resolved.

## The theory framework of the mini-program

**Fig. 1:**
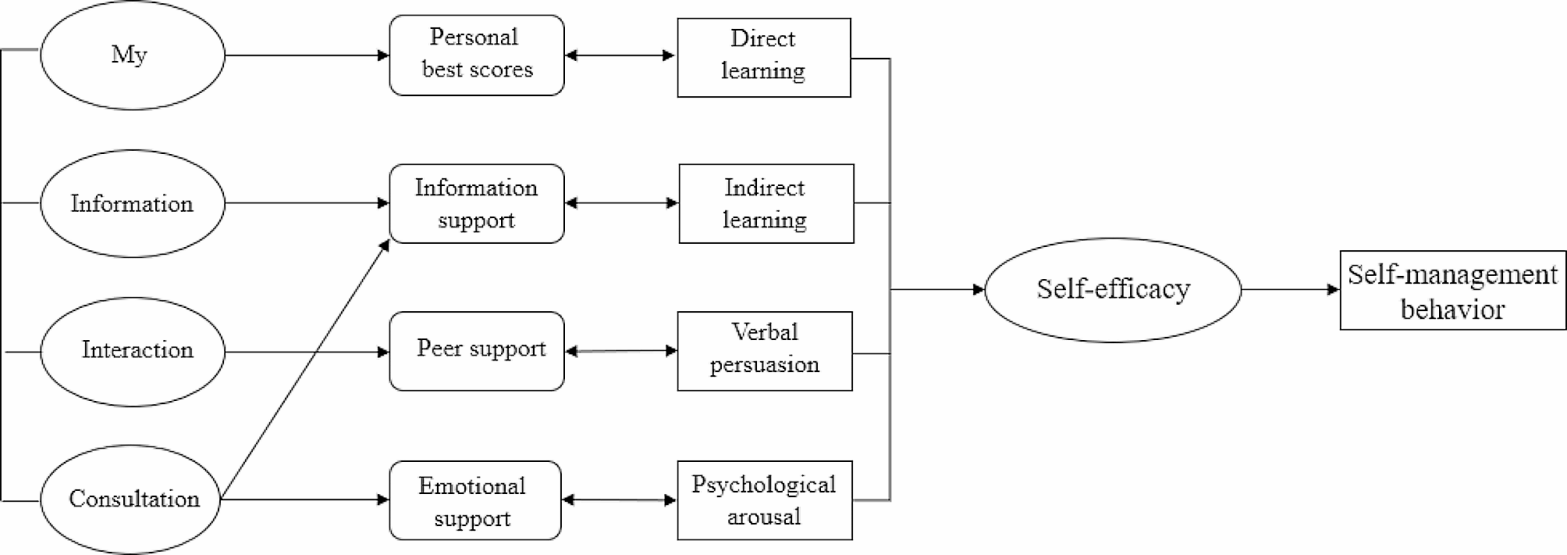
The theory framework of the mini-program

This study applied the self-management theory [37] as the theoretical framework. The self-management theory explains how individual factors and environmental factors influence an individual’s self-efficacy, which ultimately affects the generation and development of individual behaviors. Self-efficacy is influenced by direct experience, indirect learning, verbal persuasion, and psychological arousal. By providing individuals with sufficient knowledge, healthy beliefs, skills, and support, their self-efficacy is increased, and they are likely to engage in beneficial health behaviors and self-management. Individuals who are confident in their abilities to apply self-management behaviors and overcome obstacles by improving their self-management skills and persevere in their efforts to manage their health [37]. Self-efficacy is directly and linearly positively related to the active adoption of health management behaviors [38]. The functions of the various parts of the mini-program designed using self-management theory can broaden the pathways and levels of efficacy information generation in four ways: direct experience, indirect learning, verbal persuasion, and mental arousal. Patients with high self-efficacy will take positive steps to achieve desired goals and possess disease-adapted behaviors. The form of the mini-application function block diagram is shown in Fig. 1.

## Driving the development process via the contextual analysis findings

Contextual analysis [39] is a method of discerning the profound significance and influence of language, behavior, events, and so forth, by examining them within a particular environment or background. Rather than being an afterthought, contextual analysis sheds light on the meaning and inner dynamics of our primary subject of interest. Through contextual analysis, we can gain a deeper understanding of the user’s usage scenarios, including their motivations, goals, environment, and behavior. This helps us better understand user needs, as well as the problems and challenges they may encounter when using the software.

**Fig. 2:**
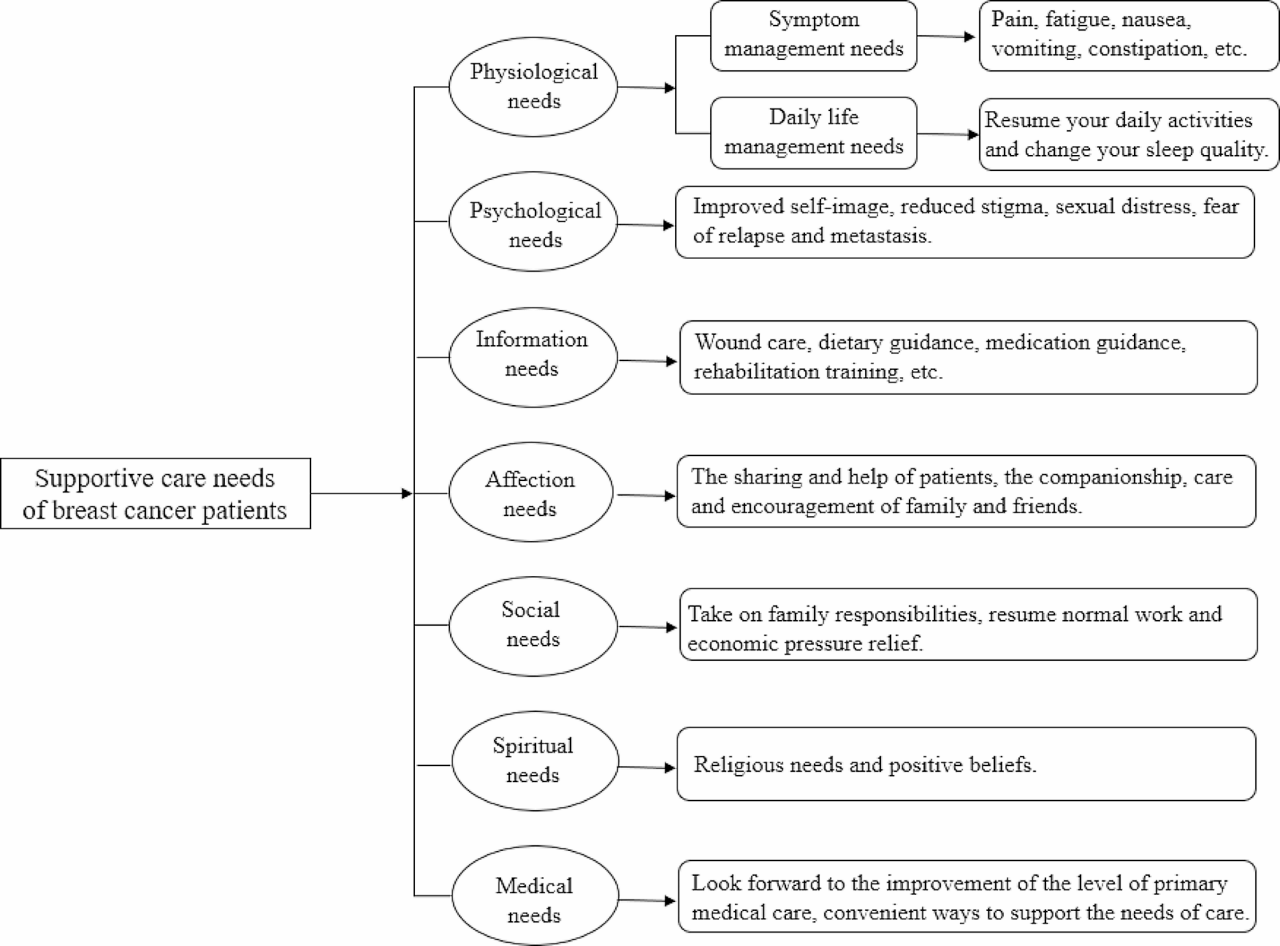
Supportive care needs of out-of-hospital breast cancer patients

In this paper, we adopted contextual analysis to gain a detailed understanding of the needs of oncology nurse specialists and out-of-hospital breast cancer patients. The research team adopted a mixed research strategy to achieve contextual analysis of the target users. A cross-sectional study was conducted among 286 patients and qualitative semi-structured in-depth interviews were applied in 12 patients to find out the supportive care needs of out-of-hospital breast cancer patients. According to the contextual analysis results from patients, the functional framework of the mini-program was constructed. See Fig. 2 for details.

Contextual analysis of breast cancer case management nurses was conducted through focus group interview. The interview results were listed as three themes: health information, personal self-management, and case management needs. Health information included breast cancer-related knowledge, the side effects of chemotherapy drugs, and symptom management measures. The key task of personal self-management contained temperature monitoring, weight management, functional exercise, and symptom management. Case management needs involved storage and management of patients’ medical records and development of a nurse-end program.

**Fig. 3:**
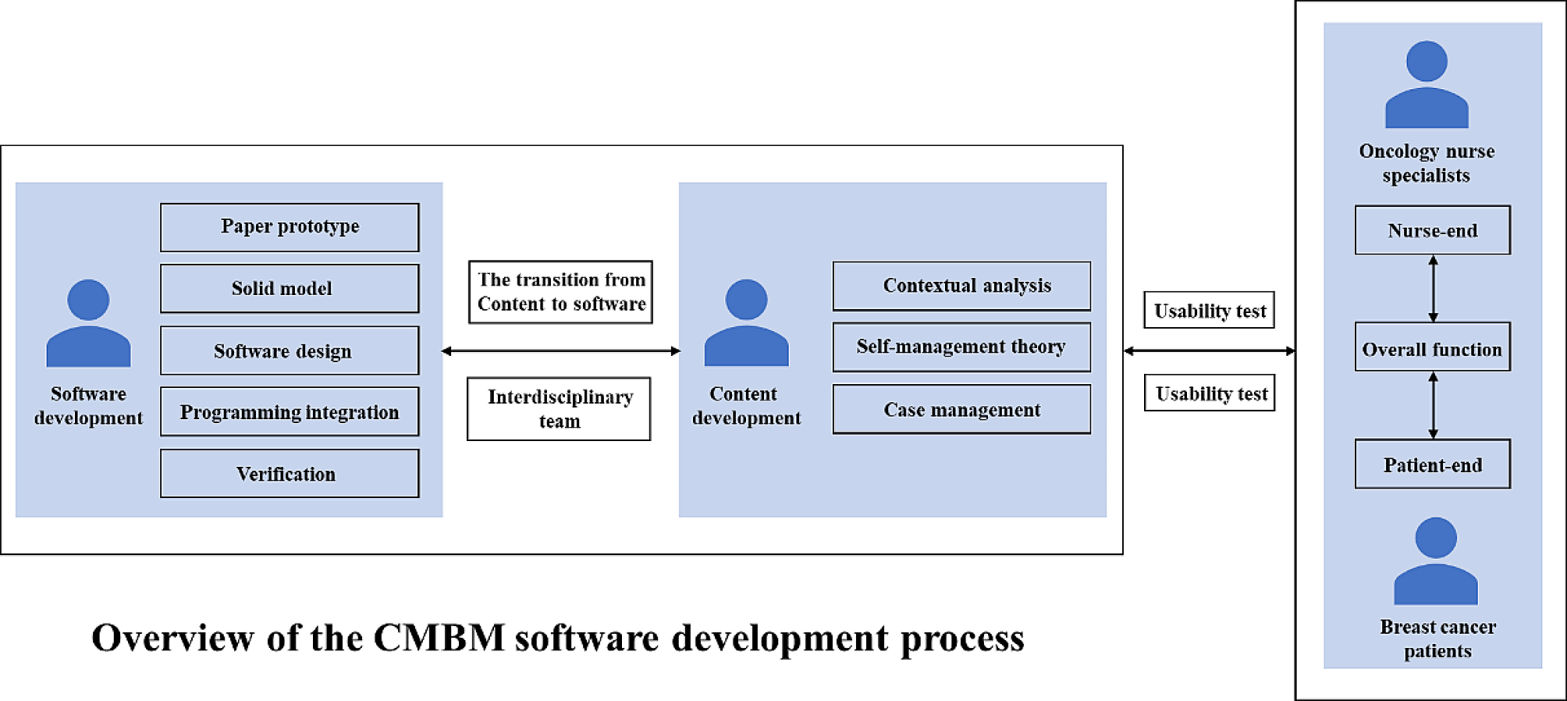
Overview of the CMBM software development process

Based on the contextual analysis results of out-of-hospital breast cancer patients and the oncology case management nurses, the framework and functional block of the mini-program were formed. An overview of the CMBM Software development process is listed in Fig. 3.

## Patient-end program functional modules

Using the results of the contextual analysis, we design the functional modules of the patient-end program based on the patient’s supportive care needs. For example, the “Information” section is designed to meet the “Information need” of breast cancer patients; the “social needs” and “spiritual needs” of patients suggest that breast cancer patients lack peer support, and for this reason, the"Interaction” section for patients has been added to the app to provide a communication platform for patients.

**Fig. 4:**
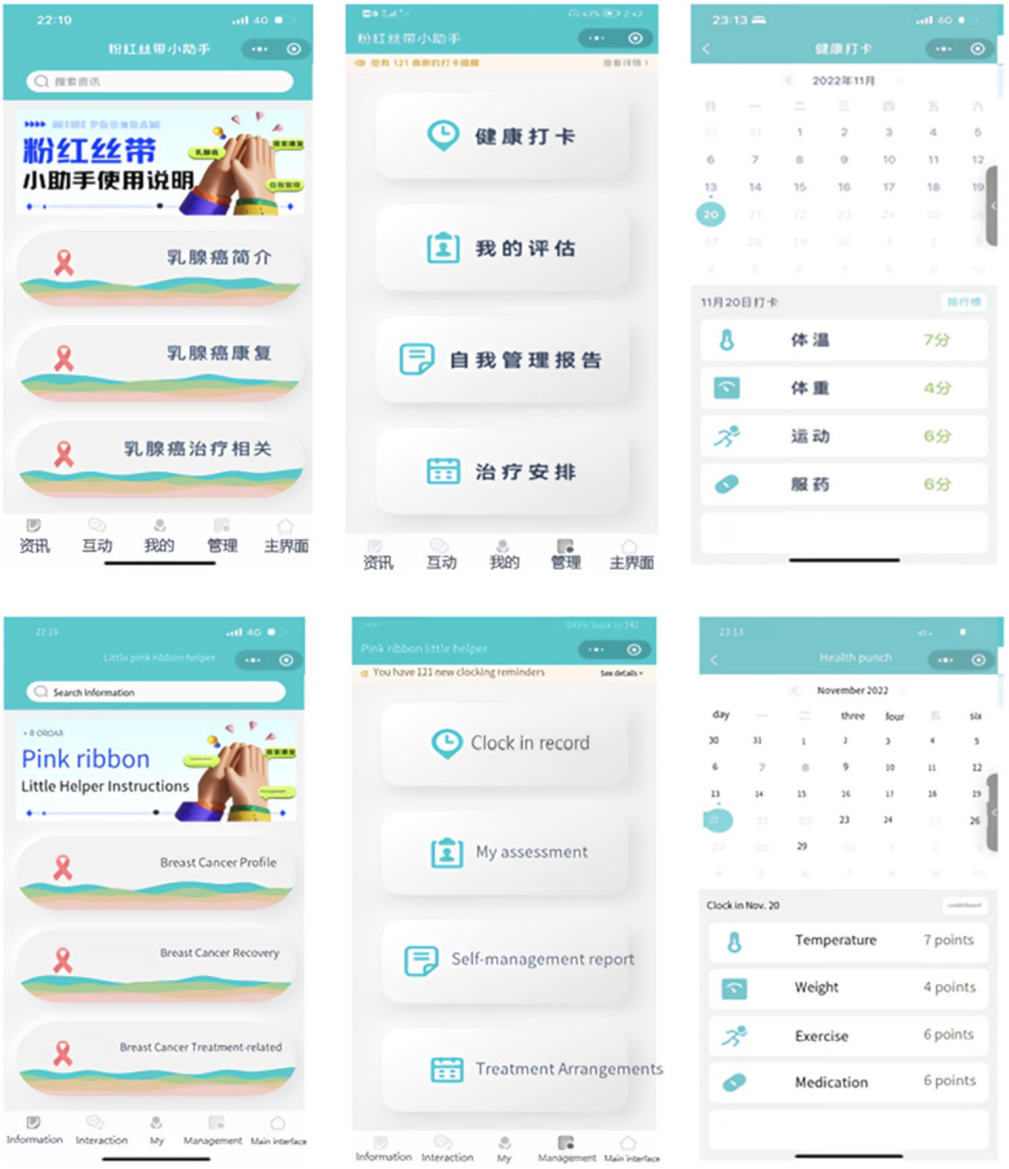
The main menu of patient-end program

The patient-end program include four functional modules: “Information”, “Interaction”, “Management” and “My”. In the “Information” module, information about breast cancer treatment and health management are compiled based on clinical guildlines. The “Interaction” module allows patients to interact with fellow patients and consult an case management nurse. In the “Management” module, patients can record and review their self-management-related health status, including three medical parameters (temperature, blood pressure, weight) and three behavioral parameters (daily steps, medication, mindfulness excersice). The “My” module enables patients to input and edit their basic personal information and medical history. The main structure and information support module contents are listed in Fig. 4.

## Nurse-end program functional modules

**Fig. 5:**
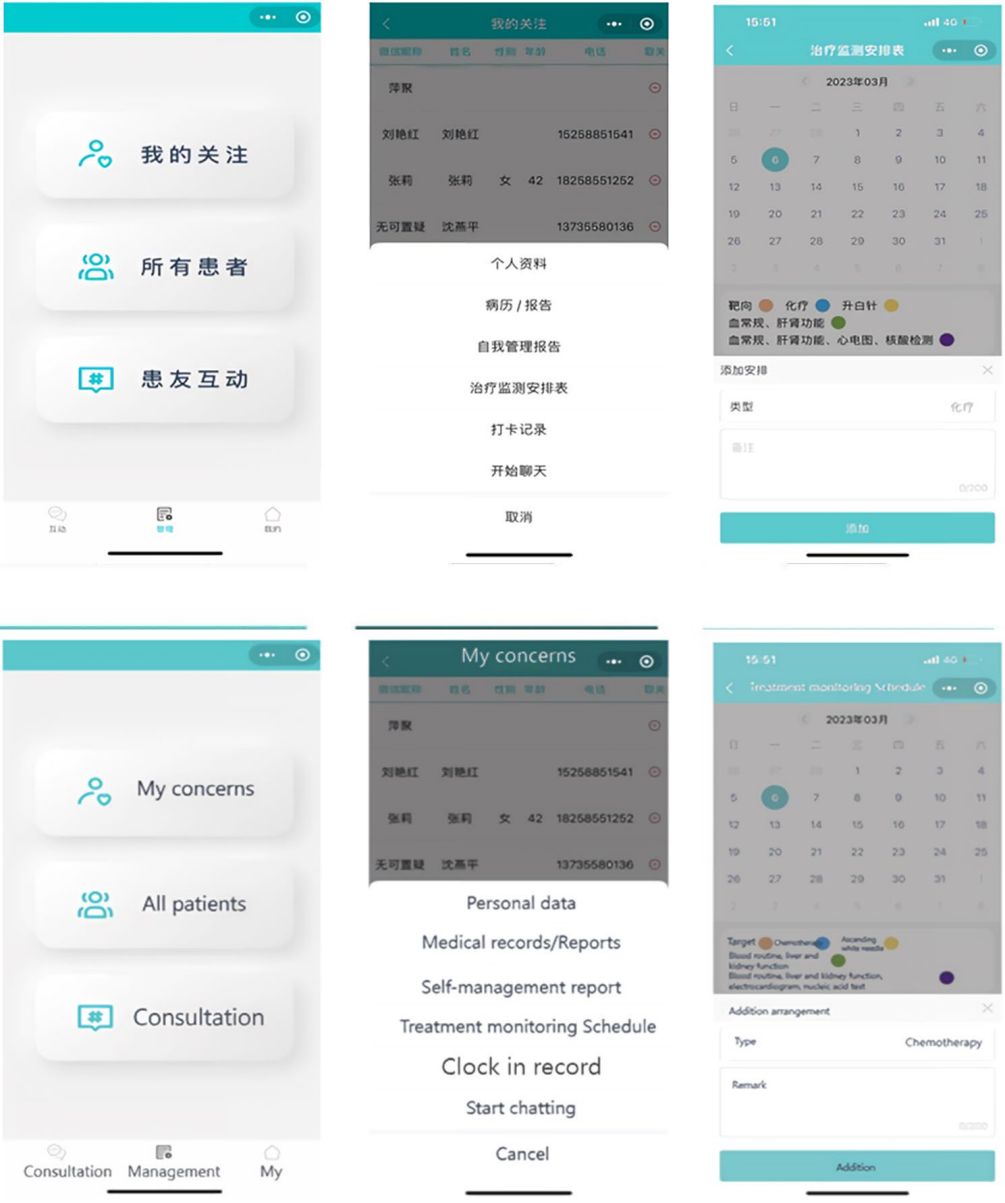
The main menu of nurse-end program

The design of the functional modules of the nurse-end program was also derived from the results of contextual analyses. The nurse-end program includes three functional modules: “Consultation”, “Management”, and “My”. The “Consultation” module is mainly used for online communication between case management nurses and patients. Nurses can enter the patient’s name in the search box to open a dialog box, and communicate with each other by sending text, voice and pictures. In the “Management” module, nurses can effortlessly search for patients by entering their name, WeChat nickname, or mobile phone number in the search box. This initiates a seamless dialogue, and with a simple click of the “+” button, patients can be promptly added to the “My Concerns” list. They can view the medical record information on its homepage, and add the postoperative treatment plan for the patient. The “self-management report” feature empowers nurses to stay up-to-date with patients’ recent well-being. By monitoring vital indicators like temperature, weight, and incidents of nausea or vomiting following chemotherapy, nurses can proactively ensure patients’ safety. The “clock in record” feature meticulously logs various patient activities including weight variations, exercise regimens, and medication adherence, providing a holistic view of their health journey. “Treatment monitoring Schedule” enables nurses to create customized chemotherapy schedules. With the first postoperative chemotherapy session scheduled in the calendar, the system seamlessly computes subsequent chemotherapy sessions and associated assessments. This transition to an online system marks a significant advancement from the traditional paper-based chemotherapy planning. Its automated scheduling and data tracking functions serve to alleviate the clinical nursing workload, enhancing efficiency and freeing up valuable time for focused patient care. The “My” module offers nurses the convenience of adding patients of interest or relevant content to their “My Favorites” section, enabling streamlined one-click access for viewing and management. The core structure and informational components of this module are outlined in Fig. 5.

## Driving the development process via the multi-stage optimization strategy

We adopted a multi-phase optimization strategy to drive the software development process. This strategy was proposed by Collins in 2005 and has become an important guiding theory for the development and evaluation of M-health interventions in recent years [40]. The strategy consists of three phases: Screening Phase, Refining Phase, and Confirming Phase. The Screening Phase need theories to identify and incorporate intervention elements. In this study, the initial version (1.0) development was based on self-management theory. Focusing on self-management, the results of contextual analysis, literature review and expert consultation were combined to design the mini-program version (1.0). The Refining Phase involves iterative adjustments to the previously version. In this study, the development team iteratively adjusted the mini-program version (1.0) according to users’ suggestions and test results. The Confirming Phase includes planning for clinical trials to test effect of the mini-program version (2.0) on self-management and recovery outcomes in out-of-hospital breast cancer patients.

## Results of system test

Eight out-of-hospital breast cancer patients were recruited for system tests. The patient’s general information is listed in Table 2.

**Table 2:** General information of patients

Number	Age	Education	Career	Chemotherapy cycles received
P1	41	Middle school	individual entrepreneur	2
P2	54	Primary school	unemployment	1
P3	43	Middle school	other occupations	2
P4	40	High school	other occupations	3
P5	32	Middle school	company employee	1
P6	43	bachelor	other occupations	6
P7	48	diploma	other occupations	1
P8	53	Primary school	other occupations	1

**Fig. 6:**
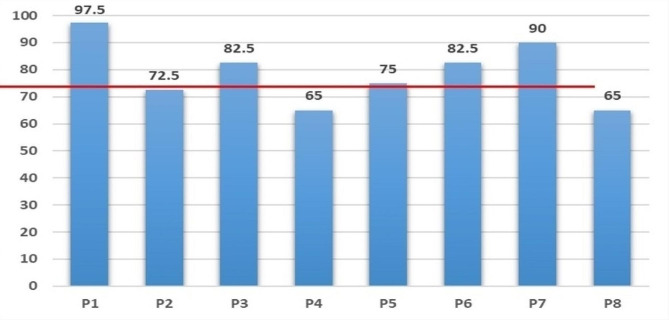
System availability scale (SUS) score of patients

The 10-item System Availability Scale (SUS)developed by Brooke was used [41]. The scale is a widely used method for quantitatively assessing user satisfaction with software systems. SUS is a Likert-5 and 10-item questionnaire (4 = strongly agree, 0 = strongly disagree), with Cronbach Alpha of 0.91. Generally, a system score above 60 on the SUS scale could be considered to be easy and simple to use, and the average score of SUS in our research is 78.75. The SUS scores of the mini-program system are presented in Fig. 6.

The research team designed the core task tests based on the typical and necessary self-management tasks of out-of-hospital patients. The core task of the “Information” module was listed as an example (Table 3). Functional tests include the passing rate for each task, and performance tests include the completion time of each task. More details can be found in Table 4.

**Table 3:** The main details of the core task test

Test module	Expected result	Test procedures
Information	Find out “Myelosuppression” in the “Information” module	(1) Click the “Information” in the home page. (2) Choose “Breast Cancer Rehabilitation” in “Information”. (3) Select “Myelosuppression” in “Physiological field”. (4) Click any tweet in the “Myelosuppression”.

**Table 4:** Task passing rate and completion time statistics

Completion time (s)	Information	Interaction	Management
Passing rate	100%	100%	100%
Minimum completion time	12	12	7
Maximum completion time	23	28	18
Mean completion time	17.1	20.3	12.8

## Discussion

In this article, we demonstrated how to create a customized software solution for breast cancer case management practices based on a multi-stage optimization strategy, applied the contextual analysis method, and followed the user-centered principle. Preliminary test results showed satisfaction and acceptance of the WeChat mini-program among both out-of-hospital breast cancer patients and oncology nursing specialists.

## Team effort

There were two typical patterns for developing M-health applications in the past. One was led by software developers, while the other was led by medical professionals. Each of these patterns has its own advantages and disadvantages. To overcome these shortcomings, some projects [42] developing M-health applications are now utilizing interdisciplinary team collaborations. This approach not only ensures the quality of the software but also makes sure that applications meet the actual needs.

In order to develop a customized software solution, our research team consisted of researchers, oncology nursing specialists, and software developers. The interdisciplinary team work dedicated to customizing software solutions together. Our team members each played to their strengths and held regular meetings to discuss and enhance our understanding and resolution of issues encountered during the software development process. Our team also included informal members: breast cancer patients, whose suggestions contributed to the practicality of the program.

## Contextual analysis and user-centered design

Contextual analysis is a valuable tool that enables developers to design systems that are more relevant and user-friendly. And it allows us to understand any context-specific characteristics, practice patterns, and the openness of the target setting’s nurses and patients towards technology [42]. User-centered design can significantly reduce the cost of program iteration. More importantly, it has a profound influence on various aspects of a program including its design, functionality, information architecture, and interactive elements [43]. By analyzing different contexts, not only did we design features that better meet user needs, but we also predicted and addressed potential issues that users may encounter when using the mini-program in advance, thereby enhancing the user experience. In the iterative development stage, we discovered and improved some deficiencies in the design through core task testing and usability testing. Notably, the completion rate of the core task test reached 100%, indicating that our application is user-friendly and easy to operate.

## Multi-stage optimization strategy

In several priority areas of public health, researchers have successfully applied multi-stage optimization strategies to enhance their work, including software development and intervention programs [44–46]. In this study, we also apply this strategy to software development. While the multi-stage optimization strategy provides an optimization framework, it is important to note that our optimization objectives (such as software functionality and content requirements) are determined by key users involved in the research (out-of-hospital breast cancer patients and oncology nurse spescialists). This project adopts a multi-stage optimization strategy, iteratively improving the development of the mini-program through screening, refinement, and confirmation stages. Each stage aims to optimize our program.

The research team plans to explore the feasibility of mini program development program through preliminary experiment, and verify the intervention effect of mini program on self-management behavior, self-efficacy and quality of life and other indicators through formal experiment. A randomized controlled trial (IRB-2020-408) was initiated in August 2022 at a Class III hospital in Zhejiang, China, and is currently in the data collection phase.

## Conclusions

There is no doubt that M-health will play a core role in the future of health care. However, to successfully implement and promote M-health applications in clinical setting, it is essential to analyze the needs of the target population. Additionally, it is crucial to determine who will be the driving force behind the implementation of the entire M-health project. This study demonstrates how to integrate M-health components into existing breast cancer case management care practices. In addition to providing a reference for other teams interested in developing and integrating M-health components into case management care models, this study also provides a reference for building M-health-featured care work models in practices.

In this study, the collaborative work of an interdisciplinary team with backgrounds in nursing and computer science, along with the active involvement of patients, not only facilitated the planning, developing, updating, and testing of M-health components based on the actual needs of the target population, but also increased the chances of acceptance and long-term implementation of the M-health program in practice.

This study demonstrates how to integrate M-health components into existing breast cancer case management practices. It provides insights for other reserch teams interested in developing and integrating M-health components into daily nursingt practice.

## Outlook

In the context of the digital age, M-health applications are rapidly becoming information sources and decision support tools for healthcare professionals and patients. However, it is crucial not to overlook the issues of information security and digital barriers for older adults.

Through interviews with outpatients with breast cancer and oncology nurses, we have gained insights into their concerns regarding information security. Some interviewees expressed concerns about information security and were worried about the risk of their personal information being leaked during app usage. Such concerns, to some extent, hinder the widespread adoption of M-health applications. Additionally, some interviewees mentioned that older patients, in general, find it challenging to learn and use the various functions of WeChat mini-programs, making it difficult to promote and apply M-health applications among the elderly population.

Solving these issues effectively is not only vital for the patients’ rights and interests but also crucial for the comprehensive implementation of M-health in practice. It is a matter that requires careful consideration in future development of M-health applications.

### Electronic supplementary material

Below is the link to the electronic supplementary material.

Supplementary Material 1

## Data Availability

The datasets generated and/or analysed during the current study are not publicly available but are available from the corresponding author on reasonable request.
