# Supplementary material for: Multi-stage optimization strategy based on contextual analysis to create M-health components for case management model in breast cancer transitional care: the CMBM study as an example
Source: BMC Nurs. 2024 Jun 6;23:385. doi: 10.1186/s12912-024-02049-x (PMC11155158; doi:10.1186/s12912-024-02049-x)
Supplement: Supplementary file 1 — Supplementary Material 1 [file 12912_2024_2049_MOESM1_ESM.docx]

**Supplementary material**

***1. Development phase of mini-program***

**1.1 Cross-sectional studies**

A total of 286 patients with breast cancer chemotherapy were included, all of whom were female, aged between 27 and 67 years, with a mean age of (49.47±8.75).

**1.2 Face-to-face interviews (patients)**

A total of 12 female cases were interviewed; their ages ranged from 33 to 59 years, with a mean age of 46.2 years. The interviewees' basic information is presented in the table S1 below.

| **NO.** | **Gender** | **Age** | **Marital status** | | **Residence** | | **Education** | **Occupation** | **monthly income（¥）** |
| --- | --- | --- | --- | --- | --- | --- | --- | --- | --- |
| A | Female | 46 | | Married | Towns | Junior high school | | Worker | 3000～5000 |
| B | Female | 33 | | Married | Towns | College | | Self-employed | ≥5000 |
| C | Female | 52 | | Married | Rural | Primary School | | Self-employed | 3000～5000 |
| D | Female | 44 | | Married | Towns | Undergraduate | | Teacher | ≥5000 |
| E | Female | 59 | | Married | Rural | Primary School | | Farmer | ≤3000 |
| F | Female | 57 | | Married | Towns | Primary School | | Unemployed | 3000～5000 |
| G | Female | 42 | | Married | Towns | College | | Self-employed | ≥5000 |
| H | Female | 49 | | Married | Rural | High School | | Worker | 3000～5000 |
| I | Female | 49 | | Married | Rural | Primary School | | Farmer | ≤3000 |
| J | Female | 53 | | Married | Towns | Primary School | | Unemployed | ≥5000 |
| K | Female | 36 | | Married | Towns | Junior high school | | Self-employed | ≥5000 |
| L | Female | 34 | | Married | Towns | College | | Employee | ≥5000 |

Table S1 Face-to-face interviews' basic information

**Interview guide A**

(1) How did you care for yourself during your recuperation at home after chemotherapy?

(2) What difficulties or problems did you encounter in caring for yourself? (Give hints based on the theoretical framework of supportive care needs: physical, psychological, informational, social, emotional, spiritual, and practical needs.)

(3) How did you cope with these difficulties or problems? What kind of help did you receive? What kind of services or help do you expect from the medical staff?

(4) In which way do you want to get these services or help?

(5) Is there anything else you would like to add?

**1.3 Focus group interviews (nurses)**

A total of seven interviewees were included in this focus group interview, covering both clinical nursing and nursing management, with a mean age of 44 ± 2.83 years and a mean length of service of 24.3 ± 5 years. The interviewees' basic information is presented in the table S2 below.

| **NO.** | **Gender** | **Age** | **Education** | **Title** | **Working experience** |
| --- | --- | --- | --- | --- | --- |
| A  B | Female  Female | 48  44 | Undergraduate  Undergraduate | Associate Nurse Practitioner | 30  26 |
|  |  |  |  | Nurse Practitioner-in-Charge |  |
| C | Female | 45 | Undergraduate | Associate Nurse Practitioner | 28 |
| D | Female | 39 | Undergraduate | Nurse Practitioner-in-Charge | 17 |
| E | Female | 45 | Undergraduate | Nurse Practitioner-in-Charge | 27 |
| F | Female | 42 | Undergraduate | Associate Nurse Practitioner | 24 |
| G | Female | 45 | Undergraduate | Nurse Practitioner-in-Charge | 18 |

Table S2 Focus group interviews ' basic information

**Interview guide B**

(1) What do you think about the design of the functional framework of the mini-program? Do you have any suggestions for this?

(2) What questions do patients ask you more often during the chemotherapy interval? What are the main self-management demands?

(3) What are your daily priorities for case management needs?

(4) Can the web-based back-office management system meet your case management needs? If not, where is it not possible? What changes need to be made?

(5) Is there anything else you would like to add?

***2. Iterative phase of mini-program***

**2.1 Face-to-face interviews (patients)**

A total of 8 patients with breast cancer were included, aged 32-54 years, with a mean age of 44.25±7.25 years; all were female. The interviewees' basic information is presented in the table S3 below.

| **NO.** | **Age** | **Education** | **Occupation** | **Chemotherapy cycles received** |
| --- | --- | --- | --- | --- |
| P1 | 41 | Junior High School | Self-employed | 2 |
| P2 | 54 | Primary School | Unemployed | 1 |
| P3 | 43 | Junior High School | Other | 2 |
| P4 | 40 | High School | Self-employed | 3 |
| P5 | 32 | Junior High School | Employee | 1 |
| P6 | 43 | High School | Other | 6 |
| P7 | 48 | College | Teacher | 1 |
| P8 | 53 | Primary School | Other | 1 |

Table S3 Face-to-face interviews' basic information

**Interview guide C**

(1) How do you feel about using the mini-program?

(2) Which features of the mini-program do you find practical?

(3) Which features of the mini-program do you find easy to use?

(4) In what ways does the mini-program help you?

(5) What do you think are the shortcomings of the mini-program? How do you suggest to improve it?

(6) Would you like to use the mini-program for breast cancer rehabilitation management after discharge? What are the reasons?

**2.2 Face-to-face interviews (nurses)**

A total of 4 nurses evaluated the mini-program's usability, with an average age of 43.5 ± 3.87 years and an average of 24.5 ± 5.57 years of work experience. The interviewees' basic information is presented in the table S4 below.

| **NO.** | **Gender** | **Age** | **Education** | **Title** | **Working experience** |
| --- | --- | --- | --- | --- | --- |
| N1 | Female | 48 | Undergraduate | Associate Nurse Practitioner | 30 |
| N2 | Female | 42 | Undergraduate | Associate Nurse Practitioner | 24 |
| N3 | Female | 39 | Undergraduate | Nurse Practitioner-in-Charge | 17 |
| N4 | Female | 45 | Undergraduate | Nurse Practitioner-in-Charge | 27 |

Table S4 Face-to-face interviews' basic information

**Interview guide D**

(1) How do you feel about using the mini-program?

(2) In what ways do you think the mini-program helps patients?

(3) What are the shortcomings of the mini-program? How do you suggest to change it?

(4) Do you recommend patients use the mini-program for disease self-management? What are the reasons?
